# Benchmarking MapRT and first clinical experience: A novel solution for collision‐free non‐coplanar treatment planning

**DOI:** 10.1002/acm2.14572

**Published:** 2025-02-05

**Authors:** Mathieu Gonod, Ilyas Achag, Jad Farah, Léone Aubignac, Igor Bessieres

**Affiliations:** ^1^ Department of Medical Physics Centre Georges‐François Leclerc Dijon France; ^2^ Sales and clinical applications, Vision RT Ltd London UK

**Keywords:** collision prediction, digital model, mapRT, non‐coplanar, treatment planning

## Abstract

In recent years, complex re‐irradiations and stereotactic treatments have triggered the use of non‐coplanar treatments for better dose conformality, entailing risks of collision between the machine and the patient, couch, or immobilization device. To ensure the plans deliverability without collisions, time‐consuming actions are typically performed, including dry runs, in‐room couch rotations, and beam configuration tests during planning. To overcome these challenges, a new tool called MapRT (VisionRT Ltd., London, UK) was developed. MapRT predicts a clearance map based on a patients' 3D model (acquired with dedicated cameras at the CT simulation) and pre‐established machine models. This work evaluates the accuracy of MapRT using a 30 × 35 × 40 cm^3^ phantom and 64 gantry/couch collision coordinates on a Truebeam Linac (Varian, Palo Alto, USA). Collision coordinates were recorded for gantry and couch rotations. The agreement of real collision coordinates and MapRT's predictions was evaluated for different buffer margins around the couch/patient models customizable in MapRT. Results of the first clinical implementation of MapRT were also reported. With no buffer margin, MapRT's predictions and experimental collision coordinates showed small average differences but with large standard deviations for gantry (‐0.5°±6.2°) and couch (‐0.1°±4.8°) collision coordinates. When excluding the kV imaging components, these values were of ‐0.8°±3.5° for gantry and 0.4°±4.4° for couch. Finally, a 3 cm buffer margin allows for 100% accurate predictions by MapRT of gantry‐to‐phantom and gantry‐to‐couch collisions. Among the ∼900 treatment plans checked with MapRT, 22 collisions could be avoided while another 6 plans still incurred a collision but these are mainly due to users' oversights. MapRT easily predict collisions in complex treatment planning. This work demonstrated its reliability using a 3 cm buffer margin. MapRT is a promising tool for increasing security, time saving and workflow improvement.

## 1 INTRODUCTION

Over the last years, major technical breakthroughs and innovations have enabled increasingly complex, precise, and efficient radiotherapy in order to allow for greater and better targeting of tumor cells while sparing healthy surrounding tissues and organs at risk.

One of the well‐established approaches involves the use of non‐coplanar beams, where different couch rotations are considered during planning, to allow higher dose conformity when compared to conventional planning.^1^, ^2^, ^3^, ^4^, ^5^, ^6^ In fact, such non‐coplanar treatments (NCTs) have first been proposed for stereotactic radiation therapy of intracranial tumors specifically for the simultaneous treatments of multiple metastasis and are now commonly used.^7^ The benefits of non‐coplanar planning have also been well documented for extra‐cranial stereotactic body radiotherapy (SBRT), lung, head and neck, and breast cancer treatments.^1^, ^2^, ^4^, ^8^, ^9^ Nonetheless, such an approach is rarely applied in routine practice.

Indeed, several drawbacks and technical limitations prevent the wide‐scale adoption of NCT delivery. The primary challenge reported by various authors remains the possible collision of the gantry/imaging device with the couch, immobilization device, or even the patient.^1^, ^9^, ^10^, ^11^ In most cases, this issue is solved by doing dry runs to confirm the absence of collisions, which requires the radiation therapist to step into the treatment room prior to applying the planned couch rotation. Considering that the NCT delivery times are considerably longer than coplanar treatments,^6^, ^7^, ^9^, ^12^ these additional in‐room verifications would further increase the session duration limiting NCT adoption. Also, the inherent risk of having the patient move due to both the longer session duration as well as the presence of the staff inside the bunker^12^ and the mechanical movement of the couch between arcs have an impact on adoption. Lastly, NCT planning could be a source of stress for the planners who could be concerned by the need for repeat planning when a collision is detected on the first day of treatment. A common practice when using NCT (even for intracranial SRS) hence involves imposing restrictions on non‐coplanar arcs limiting these to well‐known/tabulated gantry/couch angles and positions. Although such safety limits allow collision‐free planning, possible dosimetry benefits of extended arcs or couch rotations are rarely explored.

Many research and in‐house solutions have been developed to allow collision‐free treatment planning. As suggested by Dougherty et al.,^13^ existing solutions can be sorted according to whether they are based on mathematical approximation of collision‐free space,^14^, ^15^, ^16^, ^17^, ^18^ stand‐alone graphical simulation software,^19^, ^20^, ^21^, ^22^, ^23^ using scripting application programming interface (API) to build integrated 3D collision detection software^13^, ^24^, ^25^, ^26^ or using a priori patient and machine surface model to predict collision‐free space.^27^, ^28^, ^29^, ^30^, ^31^, ^32^, ^33^ Although having fairly satisfactory results, most of these solutions suffer from different limitations especially in the modeling. Indeed, some studies involved the use of simple geometries for linac modeling.^14^, ^26^, ^28^ Meanwhile, the most common modeling limitation is relevant to the accuracy of the patient and immobilization device models. Some solutions define the patient setup model only considering the body contour taken from the CT scan, which is very limiting for collision prediction.^25^, ^26^ As an improvement, several studies considered to extend the body contour beyond CT acquisition area by creating phantom based predefined extensions^24^, ^25^ or using a priori library of body surface scans.^32^ These methods still limit the accuracy of the patient model and thus the collision prediction. In some studies, full body surface images can be directly acquired with a specific infrared camera to model the patient set up geometry^28^, ^29^, ^30^, ^31^ but sometimes still with other limitations: need of a CT acquisition^31^ or complicated use of a single mobile infrared camera.^28^

A first commercial clearance mapping solution called MapRT (Vision RT Ltd., London, UK) is recently available based on precise machine modelling and patient setup geometry only generated from multiple cameras and acquisitions.^34^ The present work aims to benchmark the accuracy of MapRT (Vision RT Ltd., London, UK), through extensive experimental tests conducted on a 30 × 35 × 40 cm^3^ slab phantom positioned on a TrueBeam v.2.7 treatment delivery machine (Varian, Palo Alto, USA). The work also documents the results of the first clinical implementation of MapRT and the collision checks for about 900 treatment plans.

## 2 METHODS

### 2.1 MapRT description and potential benefits

**FIGURE 1:**
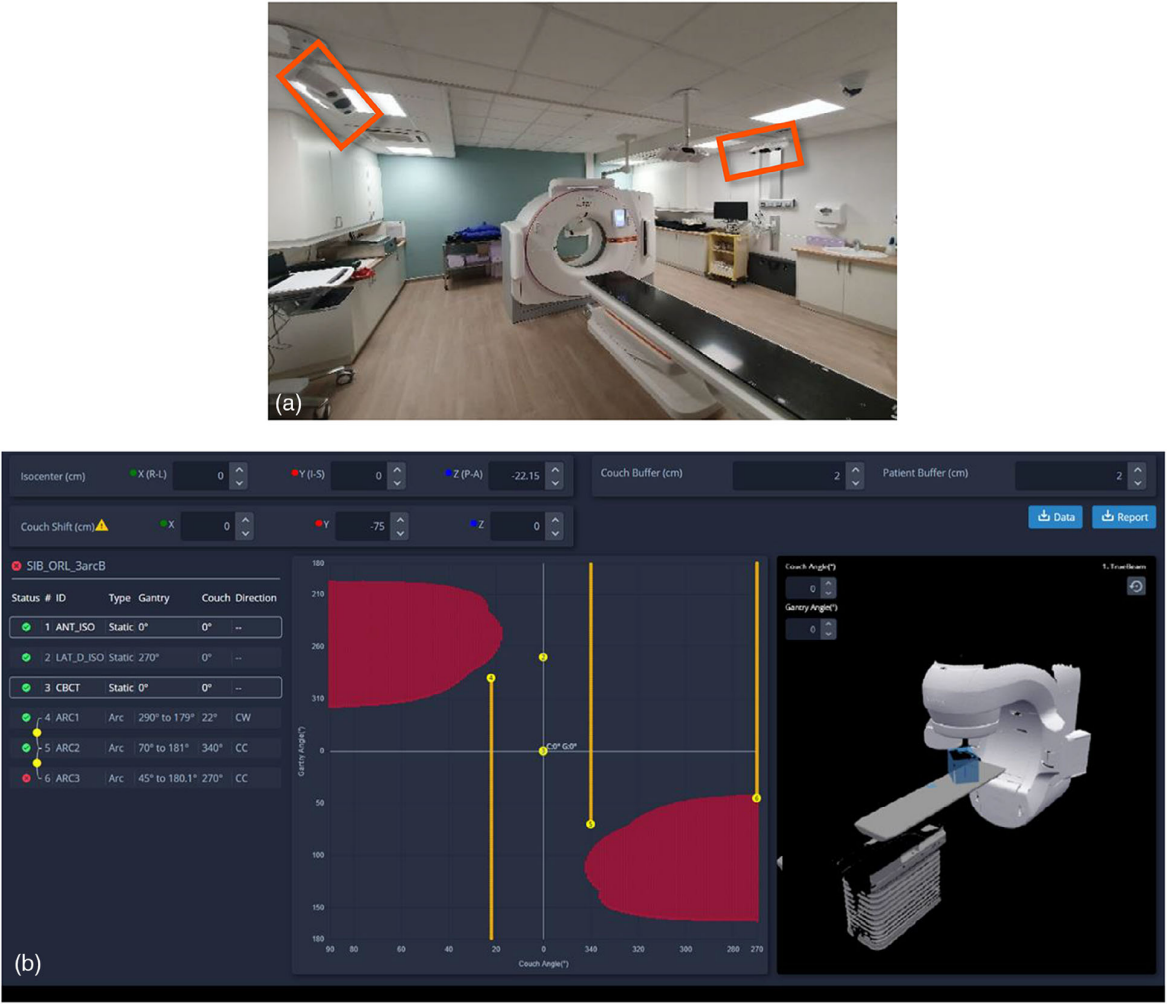
Camera setup of the MapRT system on the local Siemens Go.Sim CT (a), overview of the MapRT software interface showcasing the 3D model of the treatment room (right), the clearance map with collision areas in red and arcs/static beams in yellow (center), and collision checks results for each treatment field and for the transition between fields (left) (b).

MapRT (Vison RT Ltd, London, UK) is a novel solution for collision‐free planning that uses two Horizon cameras installed at the CT room to acquire a full body optical scan of the patient in treatment position and their individualized immobilization devices during prior or after the CT acquisition (cf. Figure 1a). Next, the acquired patient surface is automatically pushed to the MapRT browser‐based software in which a customizable model of the site‐specific Linac, treatment couch, and onboard imaging device is pre‐established. This enables the user to determine a pre‐CT clearance map allowing possible optimization of patient setup. After treatment planning, the DICOM RT Plan may be exported to MapRT to compute a clearance map for each gantry (Y‐axis) and couch angle (X‐axis) showcasing possible collisions for each treatment field/arc and each transition between fields (cf. Figure 1b). Clearance maps can also be used during treatment planning to optimize beam configurations by determining safe options for longer arcs and larger couch angles (within the 1° software resolution).

User‐defined buffers around the couch and the patient can be introduced to further increase the safety margins. Isocenter coordinates can also be edited to investigate the impact of isocenter shifts on the clearance map. Collision‐free plans with optimized arc amplitude and extent of couch motion can hence be produced, while avoiding time‐consuming dry runs.

### 2.2 Benchmarking exercise and test conditions

**FIGURE 2:**
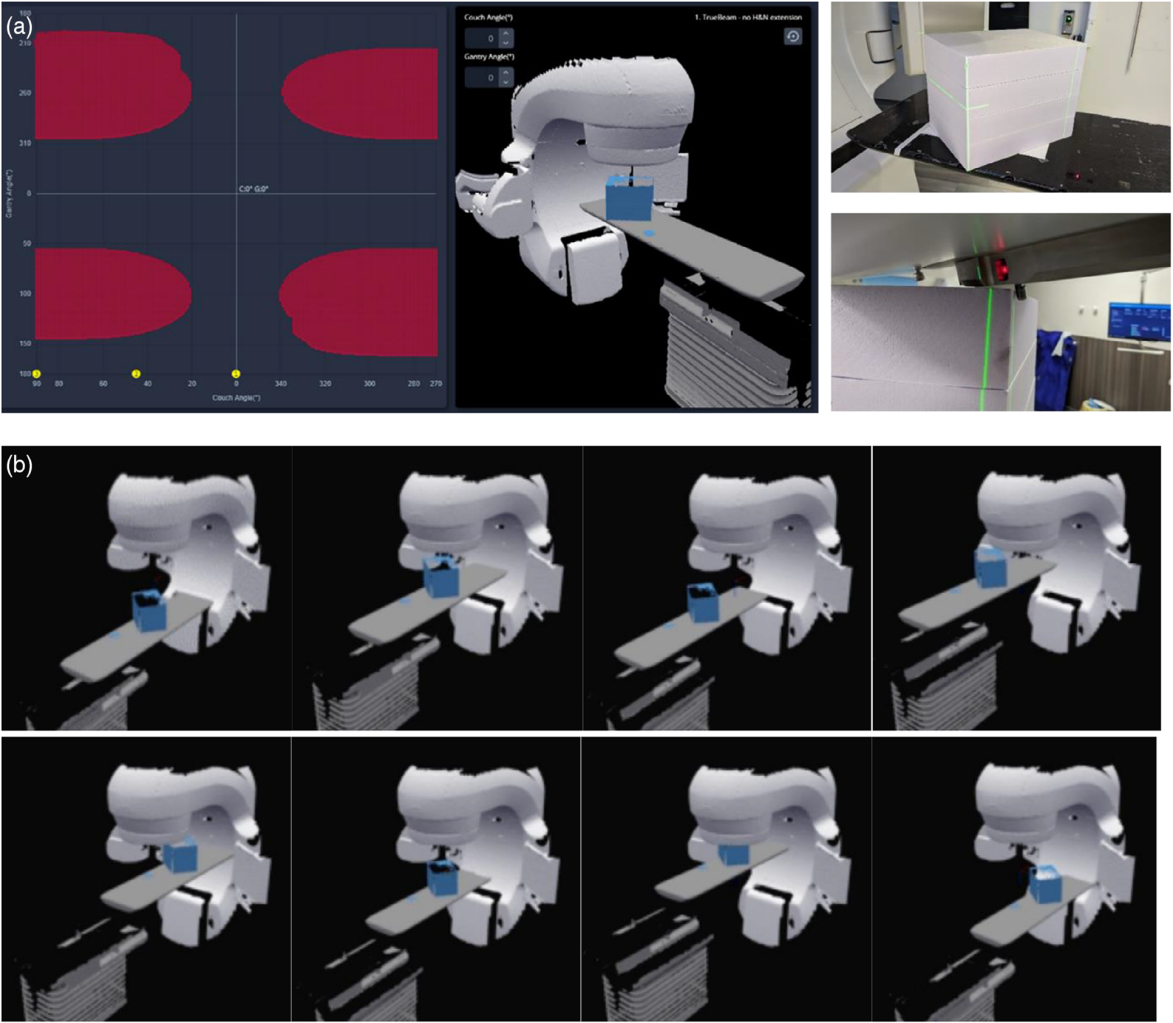
(a) MapRT clearance map of the polystyrene phantom for isocenter/couch at origin (left) and equivalent in‐room positioning (top‐right), example of an applied in‐room gantry/couch rotation to confirm the collision coordinates (bottom‐right). (b) The eighth extremal position of the phantom positioning out of the 11 achieved.

This benchmarking exercise of the MapRT software aims to evaluate the accuracy of the clearance map considering the pre‐defined model of the treatment machine and couch and using a slab phantom that is easy to model and to position with no setup uncertainties and no breathing motion as opposed to a complex anthropomorphic phantom or a human volunteer. A MapRT surface acquisition of a 30 × 35 × 40 cm^3^ slab phantom was performed (Figure 2a). The model was then added to that of a TrueBeam v2.7 linear accelerator (Varian, Palo Alto, USA) while various treatment plans were simulated using Eclipse v17 (Varian, Palo Alto, USA). Eleven different configurations of isocenter positions (Figure 2b) and couch shifts (±20 cm in lateral and vertical and ±30 cm in longitudinal) were considered to benchmark the accuracy of MapRT and its ability to anticipate any collision covering a wide extent of possible isocenter/couch positions. Next, 64 different combinations of couch and gantry angles (cf. Appendix A1) at the limit between collision/non‐collision positions in MapRT (red/black area in Figure 2a) were tested in clinical conditions. AlignRT 6.3 (Vision RT Ltd., London, UK) was used to position the phantom over the treatment couch with sub‐millimetric accuracy.^35^ Next, the gantry was first set to the expected collision position then the couch was rotated until a collision was detected. Then, the collision coordinate (i.e., couch angle) was noted. The experiment was repeated, this time by rotating first the couch to the pre‐set position then moving the gantry up until a collision was detected and the collision coordinate (i.e., gantry angle) was noted (cf. Figure 2a).

### 2.3 Collision detection analysis

To determine the accuracy of MapRT, the study compared the collision coordinates provided by the software against those measured in real conditions. The cause and position of the collision was also distinguished to determine whether it was a gantry to couch, gantry to phantom, portal imaging device (PID) to couch, kV imaging detector (kVd) to couch, or kV imaging source (kVs) to couch collision and confirm the model's accuracy within MapRT. It is worth noting that during experimental measurements, both PID and kV imaging device were parked at one of their home positions throughout these tests. Difference between MapRT and experimental collision coordinates was investigated using a Bland‐Altman test.^36^

Different clearance maps were computed considering various couch and patient buffers; these were gradually increased from 0 to 0.5, 1, 1.5, 2, 3, 4, and 5 cm. The same buffer value was applied to the couch and the patient models in MapRT. An in‐house script was developed to overlap the collision coordinates measured with a 0.1° resolution into the predicted MapRT clearance map (with a restricted 1° resolution). The script checks the collision status of the four coordinates within the MapRT clearance map that are adjacent to the measured collision coordinates and considers the coordinate to be in collision only if the four adjacent coordinates are in collision status. The script was applied to the various clearance maps to determine the minimum buffer size for MapRT to anticipate 100% of the collisions associated with the 64 couch/gantry collision coordinates.

### 2.4 Clinical implementation of MapRT

MapRT v.1.0 system was installed in September 2023 at our institution and a minor software update to v.1.1 was performed in February 2024 (bug fixes). During the first 11 months of use, and prior to final approval, each treatment plan was imported into MapRT and its collision status was checked and beam configuration optimization was considered. Initially, based on Vision RT recommendations, a 2 cm buffer was added to the patient and couch. Altogether, around 900 treatment plans were prospectively checked for collisions encompassing all cancer sites and patient setups while treatment was delivered on a Truebeam v.4.0 linac (Varian).

## 3 RESULTS

### 3.1 Collision detection accuracy

**FIGURE 3:**
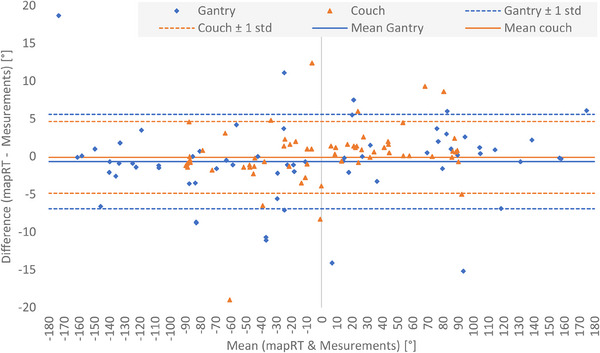
Bland‐Altman comparison of MapRT and measured collision results per gantry and couch angles for the 64 different collision coordinates.

Figure 3 plots the Bland‐Altman analysis, which compares MapRT predicted and experimentally determined collision positions and demonstrates limited average difference and quite important standard deviation for gantry (‐0.5° ± 6.2°) and couch (‐0.1° ± 4.8°) coordinates. When excluding the kV imaging components, these values were of ‐0.8° ± 3.5° for gantry and 0.4° ± 4.4° for couch. For the 64 benchmarked gantry collision positions, 31% of the differences fall within ±1°, 51% within ±2°, and 74% within ±5. These proportions were respectively of 37% (in ±1), 68% (in ±2°), and 88% (in ±5°) for the couch collision positions.

### 3.2 Minimum buffer size

Table 1 documents the percentage of equivalent collision status detected both by MapRT and experimentally as a function of buffer size. This table highlights a 4.6% correct collision prediction rate for MapRT with a 0 cm buffer and considering all possible cause of collision. Such a value increase to 64.6% with a 1 cm buffer and 98.5% with a 5 cm buffer. A 3 cm buffer around both the patient and the couch, allows for 100% of the collisions to be detected by MapRT for gantry to phantom and gantry to couch. In the case of PID to couch collisions, a 1.5 cm buffer suffices. Lastly, a 4 cm is needed for kVd to couch collisions to be fully detected while a 100% collision detection rate was never achieved for kVs to couch collisions (cf. Table 1).

**TABLE 1:** Summary of MapRT collision detection accuracy as a function of buffer size.

	Buffer (cm)	0	0.5	1	1.5	2	3	4	5
Cause	All‐to‐All^a^ (*N* = 64)	4.6%	58.5%	64.6%	73.8%	80.0%	95.4%	98.5%	98.5%
	All‐to‐Couch (*N* = 41)	4.8%	64.3%	69.0%	71.4%	76.2%	92.9%	97.6%	97.6%
	Gantry‐to‐Phantom (*N* = 23)	4.3%	47.8%	56.5%	78.3%	87.0%	100.0%	100.0%	100.0%
	Gantry‐to‐Couch (*N* = 21)	9.5%	61.9%	71.4%	76.2%	85.7%	100.0%	100.0%	100.0%
	PID‐to‐Couch (*N* = 6)	0.0%	0.0%	0.0%	100.0%	100.0%	100.0%	100.0%	100.0%
	kVs‐to‐Couch (*N* = 12)	0.0%	58.3%	58.3%	58.3%	58.3%	91.7%	91.7%	91.7%
	kVd‐to‐Couch (*N* = 2)	0.0%	0.0%	0.0%	0.0%	0.0%	0.0%	100.0%	100.0%

^a^(Gantry/PID/kVs/kVd)‐to‐(phantom/couch).

### 3.3 Clinical implementation of MapRT

Of the 900 tested plans, collisions could be anticipated and avoided thanks to MapRT for 22 plans (2.4%). For six plans (0.6%), a collision could only be detected during a dry run or at the first session. One collision was with the kVd for which different positions were not possible to test in this first version of MapRT. A second collision was due to an immobilization device that was too thin to be 3D imaged by the system. The four last collisions are due to oversights: no 3D surface acquired at the simulation or plan not imported or tested in MapRT. Excluding these particular situations, no treatment was postponed or cancelled due to collision when MapRT was used prior to treatment delivery. During the year prior to installing MapRT, up to 30 plans had to be replanned and the first treatment session postponed due to a collision detection during the dry run representing more than 2% of all the treatment plans. Lastly, it is worth noting that out of the 900 typical plans, beam configuration for 165 plans (18.4%) could be optimized based on the clearance map provided by MapRT to increase the range of arc amplitude, couch rotation, or isocenter positioning.

## 4 DISCUSSION

The present study experimentally benchmarked the first commercialized clearance mapping solution on a 30 × 35 × 40 cm^3^ slab phantom positioned on a TrueBeam V 2.7 linear accelerator demonstrating good prediction for over 64 tested gantry/couch collision coordinates. The study focused on determining the accuracy of MapRT's clearance map using the pre‐defined machine model and couch and a simple slab phantom avoiding thereby positioning errors or respiratory motion uncertainties.

Bland‐Altman analysis showed good agreement between the MapRT and experimental collision coordinates (mean values near 0°) excluding systematic bias. Additionally, no correlation was observed between the MapRT versus measurement deviation and the couch/gantry angle at which these were benchmarked. However, the standard deviation between expected and measured collision positions was rather high reaching 6.2° for gantry and 4.8° for couch coordinates (all to all scenario); these were reduced to respectively of 3.5° and 4.4° without kV imaging device. Such large standard deviation values may be explained, on the one hand, by positioning uncertainties and geometric imperfections in the MapRT models; this is particularly true for the kV imaging components parked at an unknown position (cf. Table 1). On the other hand, even small millimeter‐wide uncertainties in the 3D models in the cartesian coordinate system can induce large errors in the spherical angular coordinate system; this is particularly true for areas with low gradient in the clearance map (cf. blue area in Appendix B1). It is also worth noting that the low variability of the standard deviation value on couch collisions implies no major modeling imperfections of the treatment couch itself within MapRT.

Considering the large standard deviation, agreement on collision detection was analyzed as a function of buffer size. It was hence found that a 3 cm wide buffer around the phantom and the couch was necessary to achieve 100.0% agreement between MapRT and measured collision coordinates. This result is particularly true for the gantry to phantom and gantry to couch collision positions (even PID to couch). These results are in line with literature data for other collision management solutions. Indeed, Miao et al.^26^ and Mann et al.^25^ also found that a 3 cm buffer allows to avoid any collision. Meanwhile, Northway et al.^32^ suggested a 3 cm buffer for cranial treatments and a 6 cm buffer for lung treatments. A larger buffer of 6 cm was advised by Cardan et al.^30^ Nonetheless, most of the proposed solutions suffer from limitations in the model used, which affect the overall accuracy and reliability of the clearance map. Indeed, the modeling of the linac geometry is often simplified^14^, ^28^. For instance, Miao et al.^26^ only considered a cylindrical shaped gantry. This is also observed for patient models that are often only taken from CT images and thus limited to the CT acquisition area.^25^, ^26^ Other studies relied on the use of an infrared camera to acquire the patient surface but reported high uncertainties in the acquired patient model^28^ often leading to a larger buffer value to avoid collision.^30^ In contrast, besides offering accurate linac models, MapRT uses a full body surface acquired with four infrared cameras making it more realistic and reliable compared to most of the patient models proposed in the literature.

In the present study, lower percentages observed on the all to all (95.4%) configurations are solely due to kV imaging elements which impact the overall predictability of MapRT by 3.0% for kV detector and 1.5% for kV source. For the kV imaging source, even with the largest buffer size (5 cm) not all couch collisions could be accurately predicted (91.7%). This is likely due to the fact that kV panels may be parked at different retracted positions (and heights), whereas a single unknown position was modeled within the MapRT version used for these comparisons.

Study limitations inherent to this work include the use of an identical buffer around the phantom and the treatment couch whereas asymmetrical margins, authorized by the software, could have been applied to identify the minimal buffer size allowing MapRT to detect 100% of the collisions. Such uneven buffers could allow better identification of modeling uncertainties within MapRT (related to the Linac, couch, and imaging elements) as well as setup uncertainties and intra‐fraction motion of the patient. Another limitation is related to the experimental approach used to determine the collision gantry/couch coordinates, which consisted in sequentially fixing the gantry then moving the couch and vice versa. A simultaneous, vectorial, motion of both gantry and couch could allow more accurate experimental identification of collision coordinated; however, such motion is not allowed by the treatment machine. Nonetheless, the applied sequential approach overestimates the collision position resulting in more conservative values. In a similar way, the restricted 1° resolution in MapRT's clearance map results in a conservation overestimation of the collision coordinates. Lastly, although 64 different test conditions were checked these cannot represent all possible collision scenarios especially considering the simple 30 × 35 × 40 cm^3^ slab phantom used; anthropomorphic phantoms such as the Alderson Radiation Therapy phantom (Radiology Support Devices Inc., Long Beach, USA) could be considered in the future.

**FIGURE 4:**
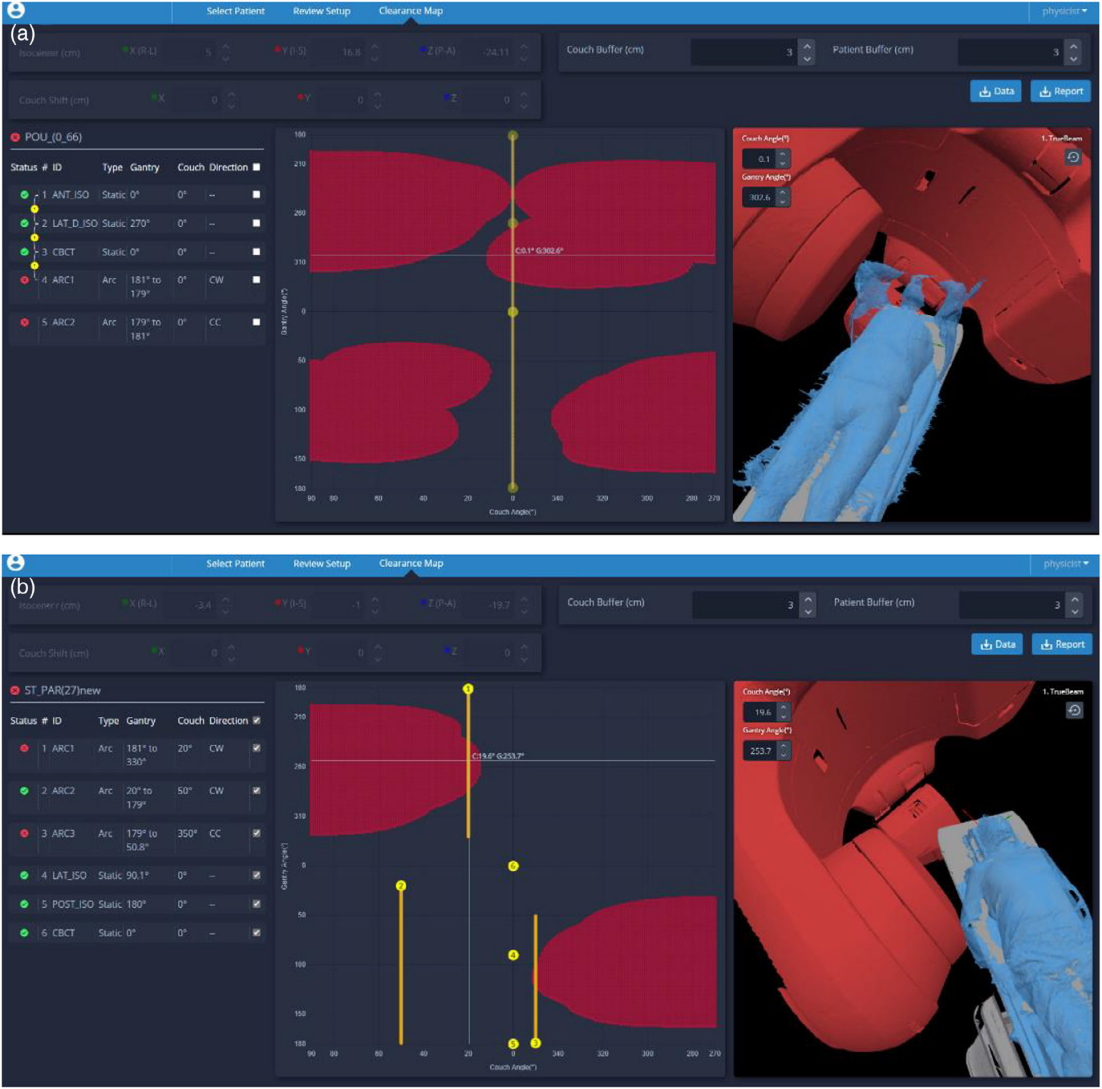
Example of clinical collisions detected thanks to MapRT: (a) lung case planned with coplanar arcs where the machine collisioning the elbow and the immobilization device, (b) cranial steretotactic planned with non‐coplanar arcs where the machine collisioning the elbow of the patient even if it is along the body.

This first report of MapRT clinical use demonstrates the reliability of the system in avoiding collisions even with a reduced 2 cm wide buffer value. Indeed, when excluding the four cases directly linked to human insights, only 2 out of 900 plans (0.2%) still encountered a collision, which was missed by MapRT and detected during dry session. One of these collisions was the result of an inherent limitation to the MapRT model of kVd positions available in the first software version (v.1.0). A newer software release of MapRT (release v.1.2) with several positions of the kV imager is now available (since July 2024) allowing for more accurate collision checks. As such, using MapRT, it is possible to safely envision removing systematic dry runs which would save precious time and increase linac availability for treatments. Meanwhile, among the 22 collision cases avoided thanks to MapRT, several collisions were observed for coplanar configurations (for instance, arms above the head and posterior isocenter cf. Figure 4), confirming that collisions are not only a concern for NCT but also all treatment plans could benefit from MapRT. Additionally, it is worth noting that our institution has a 10‐year long experience of NCT use both for intra and extracranial cancer sites. This experience probably explains the limited number of collisions detected using MapRT (22 out of 900 plans representing 2.4%) and the value of such a tool and clearance map are expected to be greater for clinics newly starting a NCT program for extra‐cranial pathologies. Finally, MapRT was also used to optimize the beam configuration for 165 plans (18.4%) in terms of rotations (gantry and couch) and arc amplitude. In the future, it could be interesting to investigate the dosimetric impact of these beam configuration optimization further emphasizing the clinical benefits of MapRT.

Lastly, the present work evaluated the first beta version of MapRT v. 1.1 highlighting a robust beta software version (no bugs or system crashes/failures whatsoever in 11+ months daily use), a simple, straightforward, and timely surface acquisition workflow, which could also be used to check if patient position at simulation is suitable for treatment. Pre‐CT clearance map calculation is also efficient (less than 1 min) and allows for patient setup optimizations. Plan verification workflow requires DICOM RT Plan export from the TPS to MapRT and less than 1 min of computation time to confirm that all fields/arcs are deliverable as well as collision‐free transition between arcs. Clearance maps allow plan beam configuration optimization with prior knowledge of gantry/couch collision coordinates. However, as a beta version, several possible improvements could be suggested such as (a) individualized modeling of one own's facility rather than using an average geometry based on lidar scans of different rooms; (b) more accurate machine modeling to include currently missing elements such as the knob in the Linac head; (c) the use of a full three Horizon‐camera‐system with optimized positioning/coverage at the CT to further improve patient/phantom surface quality; (d) availability of measurement tools/metrics within the software to verify distances and object geometry sizes; and (e) a full TPS integration of MapRT instead of manual files export. Further integration of MapRT into treatment planning systems would enable smoother and faster use of the clearance map information and to broaden beam configurations optimization strategies including the use of non‐coplanar fields. We understand that several of these improvements are being implemented by the vendor.

## 5 CONCLUSION

MapRT proved accurate and efficient in detecting 100% of the gantry or EPID collisions with the phantom or the treatment couch provided a 3 cm wide buffer is used. The 3D modeling of the onboard imaging device requires further improvement. The current local practice involves systematic use of MapRT to acquire 3D models of all radiotherapy patients, check and confirm plan delivery (even coplanar) and, for selected patients, to implement alternative non‐coplanar beam configurations allowing improved dose volume histograms and plan qualities.

## AUTHOR CONTRIBUTIONS

Mathieu Gonod: Conception and design of the work; acquisition, analysis, and interpretation of data for the work; drafting the work; final approval of the version to be published. Ilyas Achag: Acquisition, analysis, and interpretation of data for the work; final approval of the version to be published. Jad Farah: Drafting and revising the work and final approval of the version to be published. Léone Aubignac: Revising the work and final approval of the version to be published. Igor Bessieres: Acquisition, analysis, and interpretation of data for the work; final approval of the version to be published.

## CONFLICT OF INTEREST STATEMENT

The authors declare no conflicts of interest.

## Figures and Tables

**FIGURE B.1:**
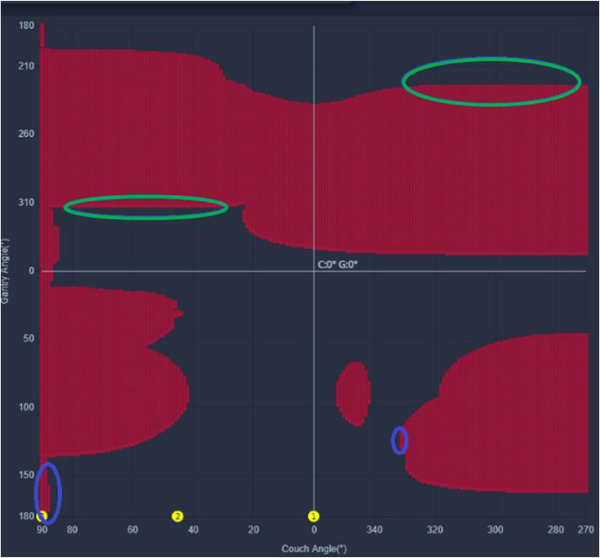
MapRT clearance maps with schematic highlights of areas with low gradients in couch angle (blue circles) or gantry angles (green circles).

## APPENDIX A

### A.1

See Table A1.

**TABLE A1:** Test conditions for the MapRT accuracy benchmarking exercise.

Phantom position			MapRT collision coordinates	
X (L‐R) (cm)	Y (A‐P) (cm)	Z (H‐F) (cm)	Couch (˚)	Gantry (˚)
−20	−20	−30	9	219
−20	−20	−30	315	252
−20	−20	−30	45	252
−20	−20	−30	351	328
−20	−20	−30	22	70
−20	−20	−30	7	84
−20	−20	−30	345	90

			**Couch (˚)**	**Gantry (˚)**
−20	20	−30	273	196
−20	20	−30	32	220
−20	20	−30	10	273
−20	20	−30	45	273
−20	20	−30	318	306
−20	20	−30	314	337
−20	20	−30	18	15
−20	20	−30	29	131

			**Couch (˚)**	**Gantry (˚)**
20	−20	−30	73	280
20	−20	−30	329	318
20	−20	−30	27	318
20	−20	−30	86	318
20	−20	−30	344	79
20	−20	−30	312	105
20	−20	−30	2	155

			**Couch (˚)**	**Gantry (˚)**
20	20	−30	23	228
20	20	−30	358	341
20	20	−30	87	341
20	20	−30	54	27
20	20	−30	316	86
20	20	−30	350	86

			**Couch (˚)**	**Gantry (˚)**
20	20	0	85	337
20	20	0	298	341

			**Couch (˚)**	**Gantry (˚)**
−20	20	0	290	17
−20	20	0	45	35

			**Couch (˚)**	**Gantry (˚)**
20	−20	0	336	275
20	−20	0	0	290

			**Couch (˚)**	**Gantry (˚)**
−20	−20	30	355	202
−20	−20	30	24	223
−20	−20	30	271	237
−20	−20	30	73	275
−20	−20	30	270	23
−20	−20	30	270	25
−20	−20	30	348	78
−20	−20	30	58	105

			**Couch (˚)**	**Gantry (˚)**
−20	20	30	273	211
−20	20	30	35	211
−20	20	30	56	332
−20	20	30	329	14
−20	20	30	13	33
−20	20	30	42	96
−20	20	30	308	158
−20	20	30	271	178

			**Couch (˚)**	**Gantry (˚)**
20	−20	30	282	235
20	−20	30	354	271
20	−20	30	27	301
20	−20	30	89	349
20	−20	30	287	78
20	−20	30	322	115
20	−20	30	338	140

			**Couch (˚)**	**Gantry (˚)**
20	20	30	24	226
20	20	30	337	243
20	20	30	340	330
20	20	30	89	0
20	20	30	275	15
20	20	30	312	157
20	20	30	89	169

## APPENDIX B

### B.1

See Figure B1.
